# Supplementary material for: Sleep quality and associated factors among adult patients with epilepsy attending follow-up care at referral hospitals in Amhara region, Ethiopia
Source: PLoS One. 2021 Dec 10;16(12):e0261386. doi: 10.1371/journal.pone.0261386 (PMC8664217; doi:10.1371/journal.pone.0261386)
Supplement: Attachment — Submitted filename: Authors response.docx [file pone.0261386.s003.docx]

**Point by point response for editors/reviewers comments**

**PLOS ONE Journal**

**Manuscript title:** Sleep quality and associated factors among adult patients with epilepsy attending follow-up care at Referral Hospitals in Amhara Region, Ethiopia

**Manuscript ID:**  PONE-D-21-26585R1

**Dear editor/reviewer.**

Dear all,

We would like to thank you for the constructive, building, and improvable comments on this manuscript that would improve the content of the manuscript. We considered each comment and clarification question of editors and reviewers on the manuscript thoroughly. Our point-by-point responses for each comment and question are described in detail on the following pages. Further, the details of changes were shown by track changes in the supplementary document attached.

**Response to Editors comment**

1. I appreciate the authors' work to revise their manuscript. This version of manuscript is significantly improved compared to the last version. I have another minor comment which should be addressed before being published. The figure one is not necessary. So, please remove this figure. Instead, you can mention the frequency and frequency percent of these reasons in the text. For example: The most frequent reason for the difficulty in maintaining sleep was pain in XX participant (59.12%), followed by midnight wakeup in XX patients (48.32%),...

**Authors’ response:** Thank you for the comments. As per your suggestion, we have removed Figure 1 and we wrote the frequencies with the corresponding percentage in text format. **(See the revised manuscript)**
